# Effects of a 12-Week Technology-Assisted Neuromuscular Training Program on Judo-Specific Fitness, Shuttle Agility, and Plantar Pressure Distribution in Adolescent Judoka: A Pilot Study

**DOI:** 10.3390/jfmk11040399

**Published:** 2026-10-01

**Authors:** Alpar Nagy, Mircea Nicolae Ordean, Vlad Teodor Grosu, Nicola Mancini, Siria Mancini, Sonia Gabriela Neagu, Emilia Florina Grosu, Daniela Aducovschi, Anca Maria Sabău, Costinel Mihaiu

**Affiliations:** 1 Doctoral School of Physical Education and Sport, Babeș-Bolyai University, 400015 Cluj-Napoca, Romania; office@activjudo.ro (A.N.); emilia.grosu@ubbcluj.ro (E.F.G.); 2 “Activ Judo” Sports Club, 405100 Câmpia Turzii, Romania; 3 Department of Physical Education and Sport, University “1 Decembrie 1918”, 510009 Alba Iulia, Romania; 4 Department of Mechatronics and Machine Dynamics, Technical University of Cluj-Napoca, 400114 Cluj-Napoca, Romania; vlad.grosu@mdm.utcluj.ro; 5 Department of Education and Sport Sciences, Pegaso Telematic University, 80143 Naples, Italy; 6 Department of Experimental and Clinical Medicine, University of Foggia, 71121 Foggia, Italy; siria_mancini.585707@unifg.it; 7 Faculty of Sciences, Physical Education and Sport, “Lucian Blaga” University of Sibiu, 550012 Sibiu, Romania; soniagabriela.neagu@ulbsibiu.ro; 8 Department of Physical Education and Sport, University of Bucharest, 010041 Bucharest, Romania; daniela.aducovschi@g.unibuc.ro (D.A.); costinel.mihaiu@unibuc.ro (C.M.); 9 Department of Physical Education, Sport and Physiotherapy, University of Oradea, 410087 Oradea, Romania; sabauancamaria@yahoo.com

**Keywords:** judo, *ippon-seoi-nage*, Special Judo Fitness Test, shuttle agility, plantar pressure, baropodometry, neuromuscular training, pilot study, *kuzushi*, adolescent athletes

## Abstract

**Background**: Judo performance is multifactorial; judo-specific anaerobic fitness and shuttle agility speed have previously been investigated separately in adolescent judoka. What remains unexplored is whether a technology-assisted neuromuscular training program can concurrently influence these established performance measures alongside plantar-support biomechanics during throwing techniques such as *ippon-seoi-nage.* Pilot data integrating these domains are lacking in adolescent judoka. **Methods**: Twenty-four male judoka (aged 15–17 years; ≥3 years of experience) were allocated to an experimental group (EG; *n* = 12) or control group (CG; *n* = 12). The EG performed a 12-week technology-assisted neuromuscular training program (BlazePod light-sensor system) before each session; the CG continued standard *uchi-komi*. Three outcome domains were assessed at baseline and post-intervention: (i) judo-specific anaerobic fitness (SJFT); (ii) specific shuttle agility (BlazePod protocol); and (iii) static plantar pressure (BTS P-WALK baropodometer). **Results**: Retention was 100% with no adverse events. A 2 × 2 mixed-design ANOVA (group × time) showed a significant interaction for BlazePod shuttle agility performance (total hits: *p* = 0.026; average sprint time: *p* = 0.009), an effect that remained significant after a sensitivity analysis controlling for baseline anthropometric imbalance between groups. A significant interaction was also observed for the SJFT index (*p* = 0.028), but this effect did not survive the same sensitivity analysis (*p* = 0.10). No significant group × time interaction was detected for any of the six static plantar pressure regions assessed (all *p* > 0.09). **Conclusions**: In this pilot study, feasibility was supported by 100% retention and the absence of reported adverse events. The data provided preliminary evidence of a group × time interaction for judo-specific shuttle agility performance that remained significant after adjustment for baseline anthropometric imbalance, a less certain interaction for anaerobic fitness, and no detectable interaction for static plantar pressure distribution. These preliminary, hypothesis-generating findings support conducting a definitive, randomized, weight-stratified controlled trial.

## 1. Introduction

Biomechanical analysis of judo highlights that effective off-balancing (*kuzushi*) depends on how the athlete distributes body weight between the feet and controls plantar pressure. Control of plantar support is therefore essential for achieving effective off-balancing, as it mediates the ground reaction force impulse that displaces the opponent’s center of mass (COM) [1,2,3].

Three-dimensional kinematic analysis of the *ippon-seoi-nage* technique reveals that the COM moves predominantly forward (≈1 m) and downward (≈19% of body height) during execution [4]. This marked anterior displacement of the COM implies a progressive transfer of body weight toward the forefoot during the *tsukuri* and *kake* phases [4,5].

Top-elite judoka generate substantially greater peak relative anterior COM velocity than sub-elite peers (2.62 ± 0.25 vs. 1.62 ± 0.28 m/s, *p* = 0.05) during the *kuzushi*/*tsukuri* phase, with no group difference in phase duration, confirming that the velocity of forward drive, underpinned by forefoot ground contact, is the key performance discriminator in *seoi-nage* [6]. Top-elite athletes also exhibit sequential proximal-to-distal angular velocity progression (hip → shoulder → arm), absent in sub-elite peers [6,7,8]. Beyond performance, technically incorrect execution, specifically, excessive external shoulder rotation of the *tsurite* arm, is associated with elbow overuse injuries, whereas athletes without pain maintain internal rotation throughout *kuzushi*, generating force along the throwing vector [9].

Modern baropodometric platforms enable standardized and reliable measurement of static and dynamic plantar pressure [10,11,12,13,14,15,16]. Neuromuscular training targeting lower-limb strength, balance, and forefoot awareness could, in principle, optimize plantar support patterns [17,18,19,20,21,22,23], although its short-term effects on static loading patterns remain uncharacterized in adolescent judoka.

Judo performance also depends on judo-specific anaerobic capacity, assessed using the Special Judo Fitness Test (SJFT) [24,25]. The SJFT requires repeated *ippon-seoi-nage* throws against two alternating *uke* across three timed bouts (15 s, 30 s, 30 s), producing a fitness index where lower values indicate superior capacity [24,25]. Lower index values reflect superior performance because the index combines throw output with post-exercise recovery heart rate; so, faster recovery alongside more throws completed improves the score.

Despite this body of biomechanical and physiological evidence, no prior study has tested a technology-assisted neuromuscular training program simultaneously targeting judo-specific anaerobic fitness, shuttle agility, and plantar pressure distribution in adolescent judoka. This is a relevant gap because adolescence represents a critical window for the consolidation of technical movement patterns, making it a period during which neuromuscular training interventions may be particularly effective at shaping long-term motor and performance outcomes.

This pilot study aimed to (1) assess the feasibility of a 12-week technology-assisted neuromuscular training program in adolescent judoka; (2) estimate effect sizes for three complementary outcome domains—SJFT anaerobic fitness, BlazePod shuttle agility, and static plantar pressure—to inform sample size calculation for a future randomized controlled trial (RCT) [26]; and (3) examine the direction and magnitude of potential anterior plantar weight transfer, considered biomechanically essential for *ippon-seoi-nage* efficiency [4,6].

Based on this rationale, we hypothesized that the 12-week technology-assisted neuromuscular training program would (i) improve judo-specific anaerobic fitness (lower SJFT index) and shuttle agility performance (BlazePod performance) in the experimental group relative to controls, and (ii) be accompanied by measurable but exploratory changes in static plantar pressure distribution consistent with a shift toward forefoot loading. The latter hypothesis is framed as exploratory given the limited evidence directly linking training-induced neuromuscular adaptations to static baropodometric outcomes in this population, as noted above.

## 2. Materials and Methods

### 2.1. Participants

Twenty-four male judoka aged 15–17 years participated. Participants were allocated to an experimental group (EG, *n* = 12; “Activ Judo” Sports Club, ACS Câmpia Turzii) or control group (CG, *n* = 12; Children’s Palace, Huedin), with club-based non-random allocation dictated by logistical constraints (proximity to the research team and club availability during the study window, rather than any systematic recruitment criterion); no club-level rationale for the observed baseline anthropometric difference between clubs was identified. All competed at regional or national level across weight categories −42 to +90 kg (Table 1). Inclusion criteria were ≥3 years of judo experience and completion of all testing. Ethics approval: Research Ethics Subcommittee of Babeș-Bolyai University (No. 19779, 9 December 2025). Written parental informed consent was obtained prior to commencement.

### 2.2. Study Design and Procedure

A quasi-experimental pretest–posttest design with non-equivalent control group was employed. Both groups trained Monday, Wednesday, and Friday (17:00–18:40) for 12 weeks (6 January–28 March 2026) during the preparatory period following a one-month training interruption. The EG performed the neuromuscular training protocol at the beginning of each session immediately after general warm-up; the CG performed standard *uchi-komi* (technique-entry) drills throughout. Baropodometric, SJFT, and BlazePod assessments were conducted at baseline (6 January 2026) and post-intervention (28 March 2026).

### 2.3. Intervention Program

The intervention comprised two sequential components. An awareness phase used a magnetic board displaying the opponent’s bilateral foot positions and eight *kuzushi* directions to reinforce the conceptual link between correct *ippon-seoi-nage* biomechanics (forward drive, forefoot support) and metatarsal-phalangeal ground contact. This phase lasted approximately 5 min per session, during which the coach verbally explained the foot positions and *kuzushi* directions displayed on the board.

A technology-assisted neuromuscular training phase used the BlazePod system (Play Pulse Ltd., Tel Aviv, Israel), with wireless illuminated pods emitting color/sound stimuli to which athletes responded in pairs [27,28]. The 12-week progressive program (Table S1, Supplementary Materials) comprised 12 exercises organized in two-week blocks, progressing from foundational semi-squats (Weeks 1–2) through isometric holds with explosive extension (Weeks 3–4), partner-resisted movements (Weeks 5–6), full partner loading and vertical lifting (Weeks 7–8), to integrated *ippon-seoi-nage* entries (Weeks 9–12). Individual effective session time ranged from 9 min 05 s to 11 min 10 s (Table S2, Supplementary Materials).

### 2.4. Measurements and Instruments

#### 2.4.1. Special Judo Fitness Test (SJFT)

Judo-specific anaerobic fitness—understood here as the integrated physiological and technical response captured by a sport-specific field test rather than isolated anaerobic capacity—was assessed with the SJFT [24,25]. The *tori* executed repeated *ippon-seoi-nage* throws against two *uke* positioned 6 m apart across three timed bouts (15 s + 10 s rest, 30 s + 10 s rest, 30 s). Total throws (SJFT_nr), heart rate immediately post-test and at 1 min (Polar H10, Polar Electro Oy), and SJFT index [(HR^final^ + HR^1-min^)/*n* throws; lower = better] were recorded [24].

#### 2.4.2. BlazePod Shuttle Agility Assessment

Specific shuttle agility was assessed with two BlazePod pods placed 6 m apart on the *tatami*. Participants wore *judogi* and performed the test barefoot, consistent with standard judo training and competition footwear conditions. A brief familiarization trial preceded the recorded assessment, after which a single timed attempt was retained as the test score. The athlete shuttled between the two pods (alternating, predictable sequence) across three bouts with timing identical to the SJFT (15 s, 30 s, 30 s with 10 s rests). Outcome variables were total hits and average sprint time per rep (ms; lower = faster). Because BlazePod served as both training tool and outcome instrument, test-familiarization effects cannot be fully excluded (see Section 4.2).

#### 2.4.3. Baropodometric Assessment

Static plantar pressure was assessed with the BTS P-WALK baropodometric platform (BTS Bioengineering S.p.A., Garbagnate Milanese, Italy; BTS G-Studio software, version 2.8.16.1). Participants stood barefoot in a standardized bilateral stance (arms at sides, gaze forward, three trials). Data were partitioned into three bilateral regions: forefoot A (metatarsal heads and toes; propulsion/*kake*), midfoot B (arch region; arch morphology), and calcaneal C (shock absorption; posterior support). Variables were coded BPM_AL/AR, BPM_BL/BR, BPM_CL/CR (L = left, R = right), expressed as % total plantar pressure.

### 2.5. Statistical Analysis

Normality was assessed with the Shapiro–Wilk test (*n* < 50 rationale [29,30]) for all predefined outcomes; full results are reported in Table S3 (Supplementary Materials). All SJFT and BlazePod variables satisfied normality (*p* > 0.05). Among the baropodometric variables, all satisfied normality except control-group post-test midfoot loading (BPM_BL: *W* = 0.852, *p* = 0.039; BPM_BR: *W* = 0.850, *p* = 0.037). Given the balanced design and the exploratory status of these two outcomes, mixed-design ANOVA was employed. For the two-group, two-timepoint design, the primary analysis for each outcome was a 2 × 2 mixed-design ANOVA (group × time), with the group × time interaction as the primary test of intervention effect; paired- and independent-samples *t*-tests are reported as supplementary within- and between-group descriptors only. Primary outcomes were the SJFT index and the two BlazePod outcomes (total hits and average sprint time), corresponding to hypothesis (i) (Section 1); the three SJFT components (throws, post-test HR, and 1 min recovery HR) were analyzed as secondary outcomes, and the six static plantar pressure regions as exploratory outcomes, corresponding to hypothesis (ii). Complete group × time interaction statistics for all outcomes are reported in Section 3.5. Because groups differed significantly at baseline in height and body mass (see Section 2.1), a sensitivity analysis was conducted using ANCOVA on change scores (post − pre) with baseline height and weight as covariates, to assess whether the observed group × time differences persisted after adjustment for this imbalance. All tests were two-tailed (α = 0.05; IBM SPSS 17.0b/Python 3.x, pingouin 0.5). Effect sizes (Cohen’s *d* for between-group change-score comparisons; benchmarks: 0.20 small, 0.50 medium, 0.80 large [31]) and 95% confidence intervals are reported as primary inferential descriptors. A preliminary planning estimate of the sample size for a future RCT was obtained with G*Power 3.1 [32] (two-tailed comparison of change scores between two independent groups, α = 0.05, power = 0.80), using the between-group change-score effect size for the SJFT index (*d* = 0.96) as input. This input is not assumed to be conservative; it derives from a small pilot sample (*n* = 12/group), in which effect sizes tend to be overestimated, and the SJFT interaction did not remain significant after anthropometric adjustment (Section 3.2). On this basis, *n* = 19 per group would be required, or *n* = 23 per group allowing for 15% attrition [*n*/(1 − 0.15)] (*N* = 46); for a moderate effect (*d* = 0.50), the corresponding figures would be *n* = 64 and *n* = 76 per group (*N* = 152). These figures are intended for planning only, and the definitive sample size should be based on the smallest effect considered practically relevant; plantar pressure did not yield a usable effect size for RCT planning purposes (see Section 3.4).

## 3. Results

### 3.1. Feasibility and Normality

All 24 participants completed the 12-week program (retention 100%); no adverse events were reported. Shapiro–Wilk tests indicated normality for all SJFT and BlazePod variables in both groups at both occasions (all *p* > 0.05). Among the baropodometric variables, only control-group post-test BPM_BL (*p* = 0.039) and BPM_BR (*p* = 0.037) departed from normality (Table S3, Supplementary Materials).

### 3.2. Special Judo Fitness Test

A 2 × 2 mixed-design ANOVA (group × time) showed a significant interaction for the SJFT index (*F*(1, 22) = 5.56, *p* = 0.028, η^2^*_p_* = 0.20; between-group difference in change scores = −1.24, 95% CI [−2.34, −0.15], *d* = −0.96); this effect did not survive an ANCOVA sensitivity analysis controlling for baseline height and body mass (*p* = 0.10; see Section 4.1). Detailed within- and between-group descriptive statistics are reported in Table 2 and Table 3. Among the secondary SJFT components, the group × time interaction was significant for post-test HR (*F*(1, 22) = 6.14, *p* = 0.021, η^2^*_p_* = 0.22) and 1 min recovery HR (*F*(1, 22) = 13.36, *p* = 0.001, η^2^*_p_* = 0.38), but not for throws (*F*(1, 22) = 1.67, *p* = 0.210, η^2^*_p_* = 0.07; Section 3.5); the 1 min HR interaction should be read in light of the significant baseline difference in this variable reported below, which makes regression toward the mean a plausible contributor.

At baseline, no between-group differences were detected for throws, SJFT index, or immediate post-test HR (all *p* > 0.45; Table 2). However, 1 min recovery HR was significantly higher in the EG than in the CG at baseline (*p* = 0.023; Table 2); this baseline imbalance was addressed via the ANCOVA sensitivity analysis reported below. At post-intervention, the SJFT index did not differ significantly between groups (14.90 ± 1.66 vs. 16.24 ± 1.81; *t*(22) = −1.893, *p* = 0.072, *d* = −0.77; Table 2), and the EG’s 1 min recovery heart rate, initially higher than the CG (*p* = 0.023), converged to near-equivalence by post-test (*p* = 0.907, *d* = −0.05); the between-group difference observed at baseline was no longer evident at post-intervention.

Within the EG, all four SJFT variables improved significantly (Table 3): throws +1.17 (95% CI [0.36, 1.98]; *d* = 0.92, *p* = 0.009); SJFT index −1.43 (95% CI [−2.23, −0.63]; *d* = −1.13, *p* = 0.002); post-test HR −4.58 bpm (95% CI [−8.82, −0.34]; *d* = −0.69, *p* = 0.037); 1 min HR −8.92 bpm (95% CI [−14.6, −3.3]; *d* = −1.01, *p* = 0.005). Three of four outcomes survived Bonferroni correction (α = 0.0125); post-test HR (*p* = 0.037) did not survive the Bonferroni-corrected threshold (α = 0.0125). No significant change was observed in the CG (all *p* > 0.27; *d* = 0.14–0.33).

### 3.3. BlazePod Shuttle Agility Test

A 2 × 2 mixed-design ANOVA showed significant group × time interactions for both BlazePod outcomes (total hits: *F*(1, 22) = 5.69, *p* = 0.026, η^2^*_p_* = 0.21; between-group difference in change scores = 2.58, 95% CI [0.34, 4.83], *d* = 0.97; average sprint time: *F*(1, 22) = 8.25, *p* = 0.009, η^2^*_p_* = 0.27; between-group difference in change scores = −299.48 ms, 95% CI [−515.75, −83.22], *d* = −1.17; Section 3.5). Unlike the SJFT finding, both effects remained significant after the ANCOVA sensitivity analysis (*p* = 0.028 and *p* = 0.009, respectively; see Section 4.2).

At baseline, EG and CG did not differ on either BlazePod outcome (total hits: *p* = 0.927, *d* = 0.04; avg sprint time: *p* = 0.423, *d* = 0.33; Table 4). The EG improved significantly; average sprint time decreased by 216.5 ms (95% CI [−383, −50]; *d* = −0.83, *p* = 0.015), and total hits increased by 2.0 (95% CI [0.1, 3.9]; *d* = 0.68, *p* = 0.037; Table 5). Average sprint time survived Bonferroni correction (α = 0.025); total hits (*p* = 0.037) did not survive the Bonferroni-corrected threshold (α = 0.025). The CG showed no change (both *p* > 0.27). At post-test, EG significantly outperformed CG on average sprint time (2334.1 ± 184.1 vs. 2557.2 ± 221.8 ms; 95% CI [−396, −51] ms; *d* = −1.09, *p* = 0.014) and total hits (*d* = 1.07, *p* = 0.016; Table 4), both surviving Bonferroni correction. This divergence is consistent with an intervention-related effect and remained significant after adjusting for baseline height and body mass (see above); however, causal attribution remains limited by the non-randomized, club-based design and potential unmeasured confounding (Section 4.5).

### 3.4. Plantar Pressure

A 2 × 2 mixed-design ANOVA showed no significant group × time interaction for any of the six bilateral plantar regions (all *p* > 0.09; region-level interaction statistics in Section 3.5; see Section 4.3).

At baseline, nominally significant between-group differences were identified for BPM_CL_Pre (*p* = 0.035, *d* = 0.92) and BPM_BR_Pre (*p* = 0.042, *d* = 0.90), though only BPM_CR_Pre survived Bonferroni correction (*p* = 0.005, *d* = 1.27, α = 0.0083), reflecting pre-existing calcaneal loading differences attributable to the anthropometric non-equivalence between clubs (Section 2.1). At post-intervention, BPM_CR_Post remained significantly different between groups (*p* = 0.004, *d* = 1.33; Table 6).

Within the EG, no pre–post changes reached significance for any plantar pressure variable (Table 7), consistent with the null group × time interaction reported above. The largest numerical change was a non-significant increase in left forefoot loading (BPM_AL: +6.13 percentage points, 95% CI [−0.8, 13.1]; within-group *d* = 0.56, *p* = 0.080), directionally aligned with the biomechanical literature on anterior weight transfer [4,6], but this within-group observation does not, on its own, support an intervention effect given the absence of a significant between-group interaction (Section 4.3). No comparable directional pattern was observed in the CG (all *d* = 0.04–0.33; Table S4, Supplementary Materials).

### 3.5. Group × Time Interactions for All Predefined Outcomes

Table 8 reports the complete group × time interaction statistics of the 2 × 2 mixed-design ANOVA for all predefined outcomes, classified as primary, secondary, or exploratory (Section 2.5).

## 4. Discussion

### 4.1. SJFT: A Positive but Fragile Finding

The mixed-design ANOVA showed a significant group × time interaction for the SJFT index (*F*(1, 22) = 5.56, *p* = 0.028, η^2^*_p_* = 0.20; between-group difference in change scores = −1.24, 95% CI [−2.34, −0.15], *d* = −0.96), driven by improvement in the EG (index: 16.34 → 14.90) relative to the CG (16.43 → 16.24). However, this effect did not survive the ANCOVA sensitivity analysis controlling for baseline height and body mass (*p* = 0.10), reflecting the anthropometric imbalance between groups described in Section 2.1. The intervention exercises (Exercises 10–12, Table S1) directly target the knee extension phase and forward hip drive identified by Ishii et al. [33] as the primary mechanism of angular momentum transfer from *tori* to *uke*; however, several intervention exercises closely replicate the SJFT throwing task, and the observed improvement may therefore partly reflect task-specific motor learning rather than purely generic anaerobic conditioning, and it cannot be dissociated with certainty from baseline group differences given the non-random allocation (Section 4.5). Future trials should include a non-specific anaerobic comparator (e.g., Wingate ergometer test) and randomized, weight-stratified allocation to disentangle these factors.

### 4.2. BlazePod Shuttle Agility

The mixed-design ANOVA showed a significant group × time interaction for both BlazePod outcomes (total hits: *F*(1, 22) = 5.69, *p* = 0.026, η^2^*_p_* = 0.21; between-group difference in change scores = 2.58, 95% CI [0.34, 4.83], *d* = 0.97; average sprint time: *F*(1, 22) = 8.25, *p* = 0.009, η^2^*_p_* = 0.27; between-group difference in change scores = −299.48 ms, 95% CI [−515.75, −83.22], *d* = −1.17), with the EG improving (total avg: 2550 → 2334 ms) while the CG did not (2474 → 2557 ms). Unlike the SJFT finding, this effect remained significant after the ANCOVA sensitivity analysis controlling for baseline height and body mass (total hits: *p* = 0.028; total avg: *p* = 0.009). However, two caveats apply. First, this is a specific linear shuttle agility test, not a visuomotor reaction-time paradigm; the stimulus was alternating and predictable, so that performance reflects explosive sprint speed and anaerobic endurance over 6 m rather than stimulus–response latency [34]. Second, the BlazePod system served simultaneously as training tool and outcome instrument, with an identical alternating protocol used in both contexts, creating a substantial risk of test-familiarization rather than generalized agility adaptation [35,36]. Future trials must employ a distinct, unpredictable-stimulus assessment tool [37].

### 4.3. Plantar Pressure: No Detectable Intervention Effect

Contrary to our a priori exploratory hypothesis (Section 1), the mixed-design ANOVA did not detect a significant group × time interaction for any of the six bilateral plantar regions (all *p* > 0.09; full results in Table 8). Mean values in the EG showed a numerically consistent pattern toward anterior weight transfer (forefoot ↑, midfoot ↓) directionally aligned with the biomechanical literature on *kake*-phase loading [4,6], but this pattern did not reach significance under the between-group analysis appropriate for this design, and the confidence intervals are wide enough to be compatible with both trivial and clinically relevant effects. We had explicitly framed this outcome as exploratory given the limited evidence linking training-induced neuromuscular adaptations to static baropodometric measures in adolescent judoka, and the present null result is consistent with that caution rather than contradicting it. Static baropodometry under quiet standing may be an insufficiently sensitive or insufficiently proximal proxy for the dynamic, explosive loading occurring during actual *ippon-seoi-nage* execution [38,39,40,41,42,43,44,45]; a future RCT should assess plantar pressure dynamically during throw execution rather than relying on static measurement.

### 4.4. Implications for Practice and RCT Design

Considered together, these pilot findings indicate that participation in a 12-week technology-assisted neuromuscular training program was associated with greater improvement in judo-specific shuttle agility performance than in the control group, with a less certain signal for judo-specific anaerobic fitness, and no detectable effect, in this pilot sample, on static plantar pressure distribution. As a preliminary planning estimate only, the between-group change-score effect size for the SJFT index (*d* = 0.96) indicates *n* = 19 per group, or *n* = 23 per group allowing for 15% attrition (*N* = 46; Section 2.5). This figure should not be regarded as conservative, because it derives from a small pilot sample, in which effect sizes tend to be overestimated, and because the SJFT interaction did not remain significant after anthropometric adjustment; for a moderate effect (*d* = 0.50), *n* = 76 per group (*N* = 152) would be required after allowance for attrition. The RCT should employ randomized allocation stratified by weight category, extend the intervention to ≥24 weeks as a pragmatic planning assumption pending further evidence on the time course of plantar-loading adaptation, and include a non-specific anaerobic comparator, an unpredictable-stimulus agility test, and dynamic plantar pressure assessment during actual technique execution.

### 4.5. Limitations

The main methodological constraints of the current study include (1) non-random, club-based allocation producing anthropometric baseline imbalance (EG significantly taller/heavier), limiting causal attribution for outcomes; because all EG participants belonged to one club and all CG participants to another, the intervention condition was completely confounded with club membership, and no statistical adjustment can fully separate the intervention effect from unmeasured coaching, environmental, or concurrent-training differences between clubs. This study should accordingly be read as a preliminary, exploratory, quasi-experimental pilot investigation rather than a controlled test of causal efficacy. This imbalance was addressed via an ANCOVA sensitivity analysis (Section 2.5), which showed that the BlazePod group × time interactions remained significant after adjustment for baseline height and body mass while the SJFT index interaction did not, underscoring the need for verified randomization in the confirmatory trial. (2) A small sample (*n* = 12/group) meant that the SJFT and BlazePod effects may have been upwardly biased (Type M error) and the plantar pressure domain was underpowered. (3) There was a training–testing overlap for SJFT (task-similar exercises) and BlazePod (same tool used for training and assessment). (4) Static baropodometry was used as a distal proxy for dynamic technique execution. (5) No kinematic measures were taken during actual *ippon-seoi-nage*, precluding direct biomechanical validation [46,47]. (6) Multiple comparisons were addressed through within-domain Bonferroni sensitivity analysis rather than a primary global correction.

Additionally, (7) no detraining follow-up precludes conclusions about durability of effects; (8) the restricted sample (male adolescents, regional/national level, Romanian clubs) limits generalizability to females, other ages, and elite international athletes. Taken together, these constraints define the agenda for the confirmatory RCT.

Three further limitations could not be resolved retrospectively with the available data and are acknowledged explicitly as follows: (9) biological maturation status was not assessed, and could have influenced anthropometric and performance outcomes independently of the intervention; (10) concurrent club-based training (volume, intensity, type, coaching) was not standardized or monitored during the intervention period and may have contributed to between-participant variability; (11) regarding testing standardization, the time of day and order of testing were not formally documented as standardized across participants at both assessment points, which cannot be verified post hoc. These limitations should be addressed prospectively in the confirmatory RCT through maturation assessment (e.g., Tanner staging or peak height velocity), training-load monitoring, and standardized testing protocols.

## 5. Conclusions

In adolescent judoka, this 12-week technology-assisted neuromuscular training program was feasible in terms of retention (100%) and the absence of reported adverse events. Participation in the program was associated with greater improvement in judo-specific shuttle agility (BlazePod) than in the control group; the group × time interaction remained statistically significant after adjustment for baseline anthropometric imbalance between groups. A group × time interaction favoring the experimental group was also observed for judo-specific anaerobic fitness (SJFT index), but this finding did not survive the same sensitivity analysis and should be interpreted with caution given the non-random, club-based allocation used in this pilot. No significant group × time interaction was detected for static plantar pressure distribution; the exploratory hypothesis of an intervention-related anterior weight-transfer pattern was not supported by the between-group analysis, though the small sample size limits the precision of this null finding.

These results should be regarded as preliminary and hypothesis-generating rather than confirmatory. They support conducting a definitive, randomized, weight-stratified RCT (provisionally *n* = 23 per group allowing for attrition; a preliminary planning estimate derived from the pilot SJFT effect size, to be refined in the trial protocol) of ≥24 weeks, incorporating a non-specific anaerobic comparator, an unpredictable-stimulus agility assessment, and dynamic plantar pressure measurement during actual technique execution.

## Figures and Tables

**Table 1:** Participant characteristics at baseline (mean ± SD).

Variable	EG (*n =* 12)	CG (*n* = 12)	Test	df	Statistic	*p*
Age (years)	15.67 ± 0.65	15.50 ± 0.67	*U*	—	*U* = 78.0	0.499
Height (cm) *	172.75 ± 9.55	164.00 ± 9.59	*t*	22	2.239	0.036
Body mass (kg) *	69.23 ± 13.67	54.90 ± 13.76	*t*	22	2.561	0.018
BMI (kg/m^2^)	23.21 ± 4.46	20.19 ± 3.25	*t*	22	1.891	0.072
Training experience (years)	3.58 ± 0.51	3.50 ± 0.52	*U*	—	*U* = 83.0	0.499

Note. Age and training experience compared using Mann–Whitney *U* (non-normal); height, body mass, and BMI with independent-samples *t*-tests. * *p* < 0.05. All participants were male.

**Table 2:** Independent-samples *t*-test results for SJFT variables at baseline and post-intervention.

Variable	Group	*n*	Mean	SD	Mean Diff.	*t*	df	*p*	*d*
Pre-intervention									
Throws Pre	Control	12	21.67	2.27	0.75	0.757	22	0.457	0.31
	Experimental	12	22.42	2.57					
Index Pre	Control	12	16.43	1.82	−0.09	−0.124	22	0.902	−0.05
	Experimental	12	16.34	1.97					
HR-fin Pre	Control	12	192.58	9.06	−0.50	−0.165	22	0.870	−0.07
	Experimental	12	192.08	5.28					
HR1min Pre *	Control	12	160.00	11.47	9.67	2.436	16.9	0.023	0.99
	Experimental	12	169.67	7.57					
Post-intervention									
Throws Post	Control	12	22.08	2.57	1.50	1.512	22	0.145	0.62
	Experimental	12	23.58	2.27					
Index Post	Control	12	16.24	1.81	−1.34	−1.893	22	0.072	−0.77
	Experimental	12	14.90	1.66					
HR-fin Post	Control	12	193.50	8.03	−6.00	−2.038	22	0.054	−0.83
	Experimental	12	187.50	6.29					
HR1min Post	Control	12	161.25	11.70	−0.50	−0.119	22	0.907	−0.05
	Experimental	12	160.75	8.69					

Note. * *p* < 0.05. *d* = Cohen’s *d*. Welch-corrected df applied where Levene’s test indicated unequal variances. Mean Diff. = Experimental − Control; positive *d* indicates a higher value in the experimental group, negative *d* a higher value in the control group.

**Table 3:** Paired-samples *t*-test results for SJFT variables, by group (pre–post comparisons).

Group	Variable	*n*	Mean Pre	Mean Post	Mean Diff.	*t*	df	*p*	*d*
Control	Throws	12	21.67	22.08	+0.42	0.923	11	0.376	0.27
Control	SJFT Index	12	16.43	16.24	−0.19	−0.498	11	0.628	−0.14
Control	HR-fin	12	192.58	193.50	+0.92	0.835	11	0.421	0.24
Control	HR1min	12	160.00	161.25	+1.25	1.142	11	0.278	0.33
Exp. **	Throws	12	22.42	23.58	+1.17	3.189	11	0.009	0.92
Exp. **	SJFT Index	12	16.34	14.90	−1.43	−3.913	11	0.002	−1.13
Exp. *	HR-fin	12	192.08	187.50	−4.58	−2.377	11	0.037	−0.69
Exp. **	HR1min	12	169.67	160.75	−8.92	−3.487	11	0.005	−1.01

Note. * *p* < 0.05; ** *p* < 0.01.

**Table 4:** Independent-samples *t*-test results for BlazePod shuttle-agility variables at baseline and post-intervention.

Variable	Group	*n*	Mean	SD	Mean Diff.	*t*	df	*p*	*d*
Pre-intervention									
Hits Pre	Control	12	26.58	2.31	0.08	0.092	22	0.927	0.04
	Experimental	12	26.67	2.10					
Avg ST Pre (ms)	Control	12	2474.1	229.8	76.4	0.816	22	0.423	0.33
	Experimental	12	2550.5	228.6					
Post-intervention									
Hits Post *	Control	12	26.00	2.59	2.67	2.621	22	0.016	1.07
	Experimental	12	28.67	2.39					
Avg ST Post (ms) *	Control	12	2557.2	221.8	−223.1	−2.681	22	0.014	−1.09
	Experimental	12	2334.1	184.1					

Note. Avg ST = average sprint time per rep (ms; lower = faster). Hits = total hits. * *p* < 0.05 (both survive Bonferroni α = 0.025). *d* = Cohen’s *d*.

**Table 5:** Paired-samples *t*-test results for BlazePod variables, by group (pre–post comparisons).

Group	Variable	*n*	Mean Pre	Mean Post	Mean Diff.	*t*	df	*p*	*d*
Control	Total Hits	12	26.58	26.00	−0.58	−0.859	11	0.409	−0.25
Control	Total Avg (ms)	12	2474.14	2557.17	+83.03	1.153	11	0.273	0.33
Exp. *	Total Hits	12	26.67	28.67	+2.00	2.370	11	0.037	0.68
Exp. *	Total Avg (ms)	12	2550.54	2334.08	−216.46	−2.869	11	0.015	−0.83

Note. * *p* < 0.05.

**Table 6:** Independent-samples *t*-test results for baropodometric variables at baseline and post-intervention.

Variable	Group	*n*	Mean	SD	Mean Diff.	*t*	df	*p*	*d*
Pre-intervention									
BPM_AL_Pre	Control	12	48.43	4.29	1.72	0.583	15.50	0.569	0.24
	Experimental	12	46.72	9.26					
BPM_BL_Pre	Control	12	16.13	8.70	−8.52	−1.711	22	0.101	0.70
	Experimental	12	24.65	14.88					
BPM_CL_Pre *	Control	12	35.45	5.66	6.82	2.254	22	0.035	0.92
	Experimental	12	28.63	8.82					
BPM_AR_Pre	Control	12	50.22	5.35	2.47	0.754	16.79	0.461	0.31
	Experimental	12	47.75	10.03					
BPM_BR_Pre *	Control	12	13.12	7.78	−10.44	−2.203	16.88	0.042	0.90
	Experimental	12	23.56	14.45					
BPM_CR_Pre **	Control	12	36.64	6.46	7.95	3.109	22	0.005	1.27
	Experimental	12	28.69	6.06					
Post-intervention									
BPM_AL_Post	Control	12	48.64	4.80	−4.21	−1.337	16.01	0.200	0.55
	Experimental	12	52.85	9.79					
BPM_BL_Post	Control	12	18.44	8.34	−2.04	−0.439	22	0.665	0.18
	Experimental	12	20.48	13.76					
BPM_CL_Post	Control	12	32.92	6.25	6.25	1.932	22	0.066	0.79
	Experimental	12	26.67	9.30					
BPM_AR_Post	Control	12	50.47	4.79	−0.57	−0.213	22	0.834	0.09
	Experimental	12	51.04	8.05					
BPM_BR_Post	Control	12	11.26	9.54	−8.23	−1.798	22	0.086	0.73
	Experimental	12	19.48	12.65					
BPM_CR_Post **	Control	12	38.28	6.99	8.80	3.265	22	0.004	1.33
	Experimental	12	29.48	6.19					

Note. A = forefoot, B = midfoot, C = calcaneal region (L/R = left/right foot); values as % total plantar pressure per foot. Mean Diff. = Control − Experimental; *d* is reported as an absolute value. * *p* < 0.05 nominally (BPM_CL_Pre and BPM_BR_Pre do not survive Bonferroni α = 0.0083); ** *p* < 0.01 (survives correction). *d* = Cohen’s *d*.

**Table 7:** Paired-samples *t*-test results for the experimental group (pre–post comparisons).

Variable (Post)	Variable (Pre)	*n*	Mean Post	Mean Pre	Mean Diff.	*t*	df	*p*	*d*
BPM_AL_Post	BPM_AL_Pre	12	52.85	46.72	+6.13	1.931	11	0.080	0.56
BPM_BL_Post	BPM_BL_Pre	12	20.48	24.65	−4.17	−1.062	11	0.311	0.31
BPM_CL_Post	BPM_CL_Pre	12	26.67	28.63	−1.97	−0.623	11	0.546	0.18
BPM_AR_Post	BPM_AR_Pre	12	51.04	47.75	+3.29	1.165	11	0.269	0.34
BPM_BR_Post	BPM_BR_Pre	12	19.48	23.56	−4.08	−1.239	11	0.241	0.36
BPM_CR_Post	BPM_CR_Pre	12	29.48	28.69	+0.78	0.603	11	0.559	0.17

Note. *d* = Cohen’s *d*.

**Table 8:** Group × time interaction statistics (2 × 2 mixed-design ANOVA) for all predefined outcomes.

Outcome	Status	Δ EG (Mean ± SD)	Δ CG (Mean ± SD)	Diff. in Δ [95% CI]	*F*(1, 22)	*p*	η^2^*_p_*	*d*	ANCOVA *p*
Judo-specific anaerobic fitness (SJFT)									
SJFT index	Primary	−1.43 ± 1.27	−0.19 ± 1.32	−1.24 [−2.34, −0.15]	5.56	0.028	0.20	−0.96	0.10
Throws (*n*)	Secondary	+1.17 ± 1.27	+0.42 ± 1.56	0.75 [−0.46, 1.96]	1.67	0.210	0.07	0.53	—
HR post-test (bpm)	Secondary	−4.58 ± 6.68	+0.92 ± 3.80	−5.50 [−10.10, −0.90]	6.14	0.021	0.22	−1.01	—
HR 1-min (bpm) ^a^	Secondary	−8.92 ± 8.86	+1.25 ± 3.79	−10.17 [−15.94, −4.40]	13.36	0.001	0.38	−1.49	—
BlazePod shuttle agility									
Total Hits (*n*)	Primary	+2.00 ± 2.92	−0.58 ± 2.35	2.58 [0.34, 4.83]	5.69	0.026	0.21	0.97	0.028
Avg ST (ms)	Primary	−216.46 ± 261.36	+83.03 ± 249.46	−299.48 [−515.75, −83.22]	8.25	0.009	0.27	−1.17	0.009
Static plantar pressure (% total)									
BPM_AL	Exploratory	+6.13 ± 11.00	+0.21 ± 4.02	5.92 [−1.09, 12.93]	3.07	0.094	0.12	0.72	—
BPM_BL	Exploratory	−4.17 ± 13.60	+2.31 ± 8.31	−6.48 [−16.02, 3.06]	1.98	0.173	0.08	−0.57	—
BPM_CL	Exploratory	−1.97 ± 10.95	−2.53 ± 7.71	0.56 [−7.46, 8.58]	0.02	0.886	0.00	0.06	—
BPM_AR	Exploratory	+3.29 ± 9.78	+0.24 ± 5.77	3.05 [−3.75, 9.85]	0.87	0.362	0.04	0.38	—
BPM_BR	Exploratory	−4.08 ± 11.41	−1.87 ± 7.16	−2.21 [−10.27, 5.85]	0.32	0.575	0.01	−0.23	—
BPM_CR	Exploratory	+0.78 ± 4.48	+1.63 ± 6.68	−0.85 [−5.67, 3.97]	0.13	0.718	0.01	−0.15	—

Note. Δ = post − pre change score (mean ± SD); Diff. in Δ = EG change − CG change; *d* = Cohen’s *d* for the between-group comparison of change scores (pooled SD). *F*, *p* and η^2^*_p_* refer to the group × time interaction of the 2 × 2 mixed-design ANOVA (with two time points, equivalent to an independent-samples *t*-test on change scores, *F* = *t*^2^). ANCOVA *p* = group effect on change scores adjusted for baseline height and body mass (sensitivity analysis, reported for the primary outcomes). Primary outcomes correspond to hypothesis (i) and exploratory outcomes to hypothesis (ii) (Section 1); secondary and exploratory outcomes are not adjusted for multiplicity and should be interpreted as hypothesis-generating. ^a^ Baseline between-group difference (*p* = 0.023; Table 2). HR = heart rate; Avg ST = average sprint time per rep (lower = faster); BPM = baropodometric region (A = forefoot, B = midfoot, C = calcaneal; L/R = left/right).

## Data Availability

The data presented in this study are available from the corresponding author upon reasonable request.

## Supplementary Materials

The following supporting information can be downloaded at: https://www.mdpi.com/article/10.3390/jfmk11040399/s1, Table S1: Neuromuscular training intervention program: exercises, sets, exercise duration, and inter-set rest by week; Table S2: Intervention program: individual work time, rest time, and total session time per week; Table S3: Shapiro–Wilk normality test results for all predefined outcomes (SJFT, BlazePod, and baropodometric variables), control and experimental groups; Table S4: Paired-samples *t*-test results for baropodometric variables in the control group (pre–post comparisons).
