# Supplementary material for: Effects of a 12-Week Technology-Assisted Neuromuscular Training Program on Judo-Specific Fitness, Shuttle Agility, and Plantar Pressure Distribution in Adolescent Judoka: A Pilot Study
Source: J Funct Morphol Kinesiol. 2026 Oct 1;11(4):399. doi: 10.3390/jfmk11040399 (PMC13692397; doi:10.3390/jfmk11040399)
Supplement: Supplementary file 1 [file jfmk-11-00399-s001.zip › jfmk-4541541-supplementary.pdf]

Supplementary Materials

Table S1. Neuromuscular training intervention program: exercises, sets, exercise duration, and inter-set rest by week.

Week	Exercise	Sets (n)	Exercise Duration (s)	Rest Between Sets (s)
1–2	1. Semi-squat	6	20	15
	2. Semi-squat with forward jump	4	40	15
	3. Semi-squat with judo-specific arm action and two forward steps	2	50	15
3–4	4. Squat with isometric hold, followed by rapid extension and vertical jump	3	40	15
	5. Squat with isometric hold, followed by forward jump	3	60	15
	6. Squat with isometric hold, followed by controlled heel lowering onto mat	1	160	30
5–6	7. Semi-squat with partner-resisted upward movement (partner holds belt)	3	50	30
	8. Forward movement with 180° turn, squat, and rapid extension	2	50	30
	9. Forward movement with 180° turn, squat, rapid extension, and forward step with pivot foot	2	50	30
7–8	10. Forward movement with 180° turn, squat between partner's feet, and vertical partner lifting	2	70	30
	11. As Exercise 10, adding three forward steps while carrying partner	2	90	30
	12. As Exercise 11, adding three partner-loaded semi-squats and throw during final extension	1	160	30
9–10	3. Semi-squat with judo-specific arm action and two forward steps	3	50	15
	9. Forward movement with 180° turn, squat, rapid extension, and forward step	2	50	30
	12. Integrated entry–lift–squat–throw sequence	1	160	30
11–12	3. Semi-squat with judo-specific arm action and two forward steps	2	50	15
	9. Forward movement with 180° turn, squat, rapid extension, and forward step	2	50	30
	12. Integrated entry–lift–squat–throw sequence	2	160	30

Table S2. Intervention program: individual work time, rest time, and total session time per week.

Week	Individual Work Time (s)	Individual Rest Time (s)	Total Time (s)	Total Time (hh:mm:ss)
1	380	180	560	00:09:20
2	380	180	560	00:09:20
3	460	120	580	00:09:40
4	460	120	580	00:09:40
5	350	210	560	00:09:20
6	350	210	560	00:09:20
7	480	150	630	00:10:30
8	480	150	630	00:10:30
9	410	135	545	00:09:05
10	410	135	545	00:09:05
11	520	150	670	00:11:10
12	520	150	670	00:11:10

Table S3. Shapiro–Wilk normality test results for all predefined outcomes (SJFT, BlazePod, and baropodometric variables), control and experimental groups.

Variable	Group	Shapiro–Wilk Statistic	df	p
BPM_AL_Pre	Control	.959	12	.776
BPM_AL_Post	Control	.976	12	.960
BPM_BL_Pre	Control	.912	12	.225
BPM_BL_Post	Control	.852	12	.039
BPM_CL_Pre	Control	.921	12	.291
BPM_CL_Post	Control	.945	12	.564
BPM_AR_Pre	Control	.885	12	.103
BPM_AR_Post	Control	.972	12	.934
BPM_BR_Pre	Control	.893	12	.128
BPM_BR_Post	Control	.850	12	.037
BPM_CR_Pre	Control	.968	12	.888
BPM_CR_Post	Control	.948	12	.612
BPM_AL_Pre	Experimental	.900	12	.158
BPM_AL_Post	Experimental	.915	12	.248
BPM_BL_Pre	Experimental	.896	12	.139
BPM_BL_Post	Experimental	.934	12	.422

Variable	Group	Shapiro–Wilk Statistic	df	p
BPM_CL_Pre	Experimental	.943	12	.534
BPM_CL_Post	Experimental	.888	12	.113
BPM_AR_Pre	Experimental	.914	12	.238
BPM_AR_Post	Experimental	.907	12	.196
BPM_BR_Pre	Experimental	.891	12	.121
BPM_BR_Post	Experimental	.898	12	.150
BPM_CR_Pre	Experimental	.905	12	.185
BPM_CR_Post	Experimental	.931	12	.396
SJFT_Throws_Pre	Control	.934	12	.421
SJFT_Throws_Post	Control	.974	12	.952
SJFT_HRfinal_Pre	Control	.906	12	.188
SJFT_HRfinal_Post	Control	.919	12	.278
SJFT_HR1min_Pre	Control	.953	12	.687
SJFT_HR1min_Post	Control	.966	12	.869
SJFT_Index_Pre	Control	.957	12	.745
SJFT_Index_Post	Control	.946	12	.577
SJFT_Throws_Pre	Experimental	.959	12	.772
SJFT_Throws_Post	Experimental	.948	12	.607
SJFT_HRfinal_Pre	Experimental	.925	12	.334
SJFT_HRfinal_Post	Experimental	.945	12	.569
SJFT_HR1min_Pre	Experimental	.911	12	.221
SJFT_HR1min_Post	Experimental	.947	12	.589
SJFT_Index_Pre	Experimental	.961	12	.802
SJFT_Index_Post	Experimental	.985	12	.997
BlazePod_TotalHits_Pre	Control	.884	12	.099
BlazePod_TotalHits_Post	Control	.981	12	.989
BlazePod_AvgSprintTime_Pre	Control	.970	12	.914
BlazePod_AvgSprintTime_Post	Control	.987	12	.998
BlazePod_TotalHits_Pre	Experimental	.957	12	.736
BlazePod_TotalHits_Post	Experimental	.935	12	.430
BlazePod_AvgSprintTime_Pre	Experimental	.876	12	.079
BlazePod_AvgSprintTime_Post	Experimental	.966	12	.869

Note. All SJFT and BlazePod variables satisfied the normality assumption ($p > .05$) in both groups at both measurement occasions. Among the baropodometric variables, only control-group post-test BPM_BL ($W = .852$, $p = .039$) and BPM_BR ($W = .850$, $p = .037$) departed from normality.

Table S4. Paired-samples t-test results for baropodometric variables in the control group (pre–post comparisons).

Variable (Post)	Variable (Pre)	n	Mean Post	Mean Pre	Mean Diff.	t	df	p	d
BPM_AL_Post	BPM_AL_Pre	12	48.64	48.43	0.21	0.181	11	.860	0.05
BPM_BL_Post	BPM_BL_Pre	12	18.44	16.13	2.31	0.963	11	.356	0.28
BPM_CL_Post	BPM_CL_Pre	12	32.92	35.45	−2.53	−1.136	11	.280	0.33
BPM_AR_Post	BPM_AR_Pre	12	50.47	50.23	0.24	0.144	11	.888	0.04
BPM_BR_Post	BPM_BR_Pre	12	11.26	13.13	−1.87	−0.905	11	.385	0.26
BPM_CR_Post	BPM_CR_Pre	12	38.28	36.64	1.63	0.845	11	.416	0.24

Note. No variable reached statistical significance (all $p > .27$). d = Cohen's d (all trivial to small: 0.04–0.33). Values expressed as percentages of total plantar pressure per foot.
